# Role of Oxidative Stress in the Pathogenesis of Non-Alcoholic Fatty Liver Disease: Implications for Prevention and Therapy

**DOI:** 10.3390/antiox10020174

**Published:** 2021-01-26

**Authors:** Johanna C. Arroyave-Ospina, Zongmei Wu, Yana Geng, Han Moshage

**Affiliations:** Department of Gastroenterology and Hepatology, University Medical Center Groningen, University of Groningen, 9713 GZ Groningen, The Netherlands; j.c.arroyave.ospina@umcg.nl (J.C.A.-O.); z.wu@umcg.nl (Z.W.); y.geng@umcg.nl (Y.G.)

**Keywords:** oxidative stress, ROS, antioxidant response, non-alcoholic liver disease, lipotoxicity, lipid metabolism, ER stress, mitochondrial dysfunction, antioxidant compounds

## Abstract

Oxidative stress (OxS) is considered a major factor in the pathophysiology of inflammatory chronic liver diseases, including non-alcoholic liver disease (NAFLD). Chronic impairment of lipid metabolism is closely related to alterations of the oxidant/antioxidant balance, which affect metabolism-related organelles, leading to cellular lipotoxicity, lipid peroxidation, chronic endoplasmic reticulum (ER) stress, and mitochondrial dysfunction. Increased OxS also triggers hepatocytes stress pathways, leading to inflammation and fibrogenesis, contributing to the progression of non-alcoholic steatohepatitis (NASH). The antioxidant response, regulated by the Nrf2/ARE pathway, is a key component in this process and counteracts oxidative stress-induced damage, contributing to the restoration of normal lipid metabolism. Therefore, modulation of the antioxidant response emerges as an interesting target to prevent NAFLD development and progression. This review highlights the link between disturbed lipid metabolism and oxidative stress in the context of NAFLD. In addition, emerging potential therapies based on antioxidant effects and their likely molecular targets are discussed.

## 1. Introduction

The maintenance of a “healthy” antioxidant status is essential for cellular homeostasis. Oxidative stress (OxS) is defined as the condition under which the generation of reactive oxygen species (ROS) exceeds the capacity of antioxidants to detoxify. The pathogenesis of several chronic diseases is related to OxS. The liver is an organ where many oxidative processes occur and is, therefore, an important target of OxS-induced damage. Oxidative stress leads to cellular dysfunction, injury, and ultimately cell death, and the impairment of the antioxidant status in the liver contributes significantly to the pathogenesis and progression of chronic liver diseases, including non-alcoholic liver disease (NAFLD) [1]. Various antioxidants have been proposed as a therapeutic agent in liver diseases, in particular non-alcoholic fatty liver disease (NAFLD), based on the clinical and experimental evidence that supports an important role of OxS in the pathophysiology of NAFLD [2]. 

NAFLD is a pathological condition characterized by fat accumulation in the liver (in more than 5% of hepatocytes) in the absence of alcohol consumption, viral infection, or drugs that can induce steatosis. NAFLD covers a wide spectrum of liver diseases ranging from simple steatosis to non-alcoholic steatohepatitis (NASH), liver fibrosis, and ultimately cirrhosis and hepatocellular carcinoma. 

Currently, NAFLD is considered the most prevalent chronic liver disease around the world with a global prevalence of 25% in the general population and an incidence of 28-86/1000 person years. Obesity is among the main risk factors for NAFLD, and NAFLD is often associated with insulin resistance, diabetes mellitus type 2, or metabolic syndrome. NAFLD prevalence is higher than 50% among patients with diabetes type 2 [3]. The accumulation of fat leads to metabolic disturbances, resulting in excessive mitochondrial ROS production and ER stress, and this, in turn, can cause inflammation, cell injury, and cell death. This mechanism is underscored by the fact that NAFLD patients often have an impaired antioxidant status, with decreased serum levels of the antioxidants vitamin E (tocopherol) and vitamin C and increased levels of lipid peroxidation products and systemic oxidative stress markers [4,5]. 

This review focuses on the mechanisms of lipotoxicity and their relation to OxS as a central driver in the pathophysiology of NAFLD. Moreover, we review the literature on the modulation of the antioxidant response as a potential therapeutic strategy. 

## 2. Antioxidant Balance in the Liver in Non-Alcoholic Fatty Liver Disease

### 2.1. Oxidative Stress Mechanisms in Non-Alcoholic Fatty Liver Disease 

Oxidative stress occurs when the balance between oxidants and antioxidants is disrupted. Oxidants or reactive oxygen species (ROS) can be classified as free radicals, which include molecules with one or more unpaired electrons or as non-radical species, generated from two free radical molecules sharing their unpaired electrons. Superoxide anions (O_2_^•−^), hydroxyl radicals (^•^OH), and hydrogen peroxide (H_2_O_2_) are major physiologically relevant ROS. Superoxide anions are generated by multiple cellular processes, and their production leads to the generation of other oxidant molecules. Superoxide dismutases (SODs) reduce O_2_^•−^ to H_2_O_2_, which in turn is converted into hydroxyl radicals (^•^OH) and water via the iron-catalysed Fenton reaction. Hydroxyl radicals directly or indirectly induce the formation of additional toxic pro-oxidants, including hypochlorous acid, peroxynitrite, and peroxyl radicals [6,7]. 

ROS are highly toxic molecules and must be detoxified by the antioxidant system. Antioxidant protection systems consist of enzymatic and non-enzymatic components. The enzymatic system includes different enzymes that detoxify ROS. Some of the most relevant are superoxide dismutases (SODs), catalase (CAT), and glutathione peroxidase and reductase (GSH-Px). Non-enzymatic components of the antioxidant system include small molecules such as glutathione, ascorbic acid (vitamin C), retinol (vitamin A), and tocopherol (vitamin E), which act as electron receptors and protect biomolecules and cell structures against damage from ROS. Other important antioxidants include heme oxygenase-1 (HO-1) and redox proteins [1,6].

In NAFLD, there are many sources of OxS. Oxidative phosphorylation (OxPhos) in mitochondria generates ATP, and superoxide anions (O_2_^•−^) are generated as a by-product of OxPhos. Therefore, OxPhos is an important source of OxS. Increased β-oxidation inside mitochondria and peroxisomes is an additional source of ROS.

In addition to mitochondria, the endoplasmic reticulum (ER) is a source of ROS due to cytochrome P450 (CYP) activity and/or microsomal metabolism or via increased expression of CHOP. It is important to mention that ROS are functional molecules that can also modulate cell signalling and the cellular response to stress [8]. Finally, the inflammatory response also contributes to OxS [9]. During liver injury, OxS induces the activation of redox-sensitive transcription factors, such as NF-kB, Egr-1, and AP-1, leading to an inflammatory response and the activation of cell death pathways in hepatocytes [10,11].

An important component of the antioxidant system is OxS-induced transcription. The nuclear factor E2-related factor 2 (Nrf2) is a redox-sensitive transcription factor and the major regulator of the redox balance. In normal conditions, it is present in the cytoplasm bound to the cytoskeletal-anchoring protein Kelch-like ECH-associated protein 1. High levels of ROS lead to the release of Nrf2 and its translocation to the nucleus to promote the transcription of antioxidant genes, which are regulated by antioxidant response elements (ARE) [12]. Among others, Nrf2 regulates glutathione levels and maintains the reduced glutathione/oxidized glutathione ratio (GSH/GSSG). Moreover, Nrf2 controls the expression of many detoxifying enzymes that eliminate molecules such as H_2_O_2_ and peroxide radicals from the cytosol, mitochondria, and the ER [13]. Interestingly, Nrf2 expression appears to be modulated by a variety of inducers (both endogenous as well as exogenous), such as electrophilic agents, redox-active compounds, and xenobiotics [14,15]. Genes that are positively regulated by the Nrf2 pathway include detoxifying enzymes (Phase I, II, and III), redox proteins involved in GSH-based antioxidant mechanisms, and genes related to lipid metabolism [13]. 

Nrf2 is also involved in lipid metabolism and may play a role in the protection against liver damage during the development of steatosis and steatohepatitis [16]. Nrf2 is important for mitochondrial homeostasis, and it has been demonstrated that Nrf2 activation is necessary to maintain mitochondrial integrity [17] and can directly affect the efficiency of mitochondrial fatty acid oxidation [18]. With regard to lipid accumulation, it has been suggested that Nrf2 represses the expression of key enzymes involved in fatty acid synthesis, thus alleviating hepatic steatosis [19]. Additionally, some in vivo studies using high fat diet (HFD) models of NAFLD showed a negative correlation between Nrf2-induced transcription and hepatic lipogenesis, suggesting that Nrf2 may decrease fatty acid synthesis and lipid accumulation [20,21]. On the other hand, there are also controversial studies that failed to detect an effect on lipid metabolism and even reported that Nrf2 activity increases lipid accumulation [22,23]. 

The role of Nrf2 in NAFLD is complex and not fully understood yet. Nevertheless, Nrf2 has been proposed as a potential therapeutic target in NAFLD. Nrf2-deficient mice (Nrf2^−/−^ KO) challenged with methionine- and choline-deficient (MCD) diet show exacerbation of liver inflammation and steatosis compared to control mice (Nrf2^+/+^) [24]. Additionally, Nrf2^−/−^ KO mice, before the start of the MCD diet, showed an altered lipid metabolism, an increased expression of cytochrome P450 enzymes, and lower levels of glutathione, but normal glucose metabolism. Interestingly, in this model, the MCD diet induced an antioxidant response in normal mice but significant oxidative stress in Nrf2^−/−^ KO mice, confirming that OxS, together with impairment of lipid metabolism, is sufficient to drive NAFLD progression even in the absence of insulin resistance. Likewise, restoration of the Nrf2 pathway in Nrf2^−/−^ KO mice improves the fatty liver phenotype [25] mainly by increasing the hepatic antioxidant response and by modulating the expression of lipid-metabolism-related genes such as PPAR α and SREBP1c. 

The Nrf2 pathway might be involved in the protection of the liver against OxS and in the pathophysiology of NAFLD. Furthermore, the role of the Nrf2 pathway in lipid accumulation seems to be highly context-dependent and needs further elucidation. Taking into account the importance of the triglyceride/free fatty acid (TG/FFA) balance in NAFLD and the potential role of Nrf2 in modulating this balance, the Nrf2 antioxidant response might be related to an increase in TG synthesis as a protective mechanism. The role of Nrf2 in NAFLD patients is not completely elucidated yet, and its role in disease progression and its potential as a therapeutic target remain unclear. Therefore, studies to evaluate Nrf2 activating compounds for the prevention and treatment of NAFLD are important, and we will discuss some of these novel compounds that are currently being investigated as potential therapies in NAFLD. 

The NF-kB pathway has been proposed as a therapeutic target in chronic liver diseases due to its role in OxS-mediated responses [26]. Activation of the NF-kB pathway can mediate protective mechanisms in conditions of OxS, e.g., via reducing ROS generation and the induction of autophagy [27]. Additionally, NF-kB promotes the expression of antioxidant genes such as MnSOD, Glutathione S transferase, and NADPH dehydrogenase [28]. On the other hand, it has been demonstrated that NF-kB signalling is related to both pro-oxidant as well as antioxidant effects and that it is related to the activation of the ER stress response [29]. NF-kB is a redox-sensitive transcription factor, and ROS may modulate its activation through the induction of the pro-inflammatory cytokine TNF-α, while antioxidants such as NAC (*N*-acetyl-l-cysteine) prevent NF-kB activation [30]. 

Furthermore, an interaction between NF-kB and the Nrf2 pathway also exists. In general, it has been reported that NF-kB negatively modulates Nrf2 transcription by competition with CBP (CREB-binding protein)–p300 complex [31], and Nrf2 deficiency (knockout) is associated with increased NF-kB activation [32]. Likewise, it has been reported that Nrf2 activation, either in vitro or in vivo, by different antioxidants, prevents inflammation via inhibition of the NF-kB pathway [33,34]. 

In the context of NAFLD, it has been demonstrated that the loss of Nrf2 leads to hepatic insulin resistance via an NF-kB dependent mechanism [35], showing the pivotal role of OxS and NF-kB-mediated inflammation in the onset of insulin resistance. Likewise, other studies have shown that activation of the Nrf2 pathway, with consequent NF-kB inhibition, improves insulin sensitivity in HFD-fed rats [36]. 

Modulation of NF-kB signalling by antioxidants has been suggested as a potential therapeutic target in NAFLD, also due to its anti-inflammatory properties. Recent reports show that antioxidant treatment in MCD-induced NASH mice leads to the induction of Nrf2 target genes and the suppression of the NF-kB signalling pathway, resulting in the amelioration of hepatic steatosis, fibrosis, and inflammation [37]. NF-kB modulation independent of Nrf2 by antioxidants such as green tea catechins has also been reported to improve NAFLD [38] by decreasing gut-derived LPS, which decreases inflammation in the liver. 

These findings suggest crosstalk between NF-kB and Nrf2 as a promising therapeutic target for NAFLD via inhibition of OxS related signalling.

### 2.2. Oxidative Stress Biomarkers in NAFLD Patients

Several studies have reported an impaired redox status in the majority of NAFLD patients, as indicated by increased levels of OxS markers and lipid peroxidation products in serum/plasma. 

In this regard, other oxidative stress markers have been studied in serum/plasma and liver samples from NAFLD patients, and increased levels/activity for most OxS markers have been reported, such as 8-isoprostane, 8-OH-dG, and TBARS/MDA. NAFLD patients also show increased levels of peroxidised lipids, such as malondialdehyde (MDA) and 4-hydroxynonenal (4-HNE), which are often used as biomarkers of lipid peroxidation in clinical practice [9]. Moreover, a recent report suggests that reduced levels of free thiol plasma levels can be considered as a global marker of the systemic load of reactive species and can be used as a biomarker for NAFLD [39]. 

Although most studies show decreased levels of hepatic antioxidant enzymes in NAFLD patients [2], some studies reported conflicting results, showing both increased as well as decreased serum levels of antioxidant enzymes such as SOD, GPx, and GSH in NAFLD patients [40,41]. These conflicting observations could be explained by the pathophysiology of NAFLD in which an initially adaptive antioxidant response to excessive ROS production is followed by exhaustion of the antioxidant system, resulting in lower levels of antioxidant enzymes. Furthermore, a cross-sectional study showed that a high proportion of NAFLD patients had low levels of dietary antioxidants such as vitamin C and retinol, and lower intakes of vitamin A and vitamin E as well [42]. 

Additional studies comparing the redox status in serum/plasma versus liver tissue at different stages of NAFLD/NASH will be informative to clarify the role of antioxidant components as biomarkers of progression of NAFLD/NASH and their potential as therapeutic targets. 

## 3. Lipotoxicity and Oxidative Stress 

**Figure 1:**
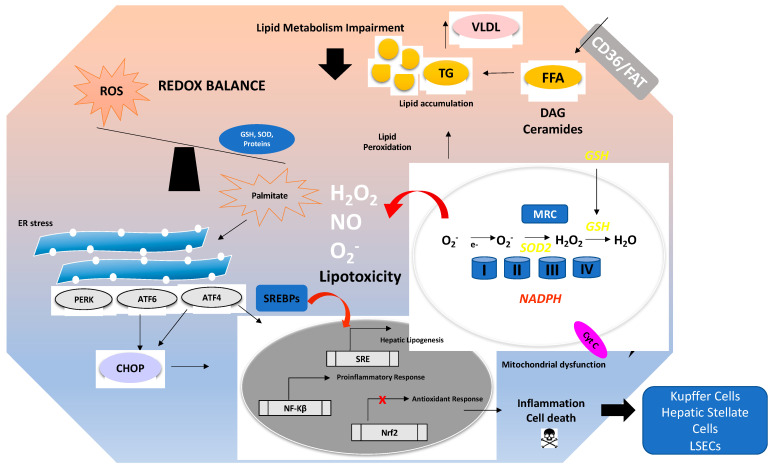
Overview of oxidative stress and antioxidants in the context of non-alcoholic fatty liver disease (NAFLD). The pathogenesis of NAFLD is a multifactorial process involving several mechanisms, ultimately leading to a disturbed redox balance. Impairment of lipid metabolism, e.g., by excessive dietary intake of fat and carbohydrates, leads to steatosis. This can be aggravated by insulin resistance. Free fatty acids (imported by, e.g., CD36 or FAT) such as palmitate cause lipotoxicity by increasing the levels of toxic lipid species such as ceramides and diacylglycerols (DAG). Mitochondrial dysfunction, impairment of β-oxidation, and endoplasmic reticulum (ER) stress can all increase the generation of ROS leading to lipid peroxidation. ER stress induces the UPR, i.e., Unfolded Protein Response. Sustained ER stress and sustained activation of the UPR will trigger activation of the ER stress proteins PERK, ATF6, ATF4, and CHOP, leading to a proinflammatory response and activation of cell death pathways in hepatocytes. ER stress might lead to the activation of sterol regulatory element-binding protein 1C (SREBP1c) and further translocation to the nucleus, thus promoting hepatic lipogenesis. The impaired redox balance also affects the antioxidant response (e.g., the Nrf2 pathway) and leads to decreased levels of antioxidants (e.g., GSH and SOD).

There are many mechanisms involved in the development and progression of NAFLD. A schematic overview of these mechanisms is depicted in Figure 1. Excess free fatty acids (FFAs) induce lipid accumulation leading to steatosis and a general impairment of lipid metabolism. In addition, insulin resistance and diabetes type 2 can develop and aggravate the consequences of excess lipid accumulation. 

In NAFLD, there is an imbalance in the distribution of different types of lipids between liver cell types, resulting in inflammation triggered by non-parenchymal cells (mainly Kupffer cells), which ultimately leads to hepatocellular damage and fibrogenesis. Indeed, we have previously shown that extracellular vesicles released from steatotic hepatocytes induce an inflammatory response in Kupffer cells and a fibrogenic response in hepatic stellate cells [43]. Direct effects of free fatty acids on Kupffer cells include M1 polarization leading to a pro-inflammatory phenotype [44,45]. Lipotoxicity, defined as a harmful effect of lipid accumulation in non-adipose tissue, is considered a central mechanism in NAFLD progression [46]. The main targets of this lipotoxicity are ER; mitochondria; and signalling pathways, such as JNK and NF-κB, related to inflammation and cell death [47]. Lipotoxicity is to a large extent mediated by OxS phenomena in the liver and will be discussed in the subsequent sections. 

Within hepatocytes, FFAs such as palmitate induce lipotoxicity either directly or by increasing the levels of deleterious lipid species such as ceramides and diacylglycerols (DAGs) [48]. Ceramides impair fatty acid oxidation and induce mitochondrial dysfunction [49]. DAGs can activate NF-κB and/or protein kinase C (PKC). Increased lipid peroxidation, impairment of β-oxidation in mitochondria, and the induction of an ER stress response also aggravate the direct toxic effects of lipids [50,51]. The role of ER stress and the Unfolded Protein Response (UPR) in NAFLD is discussed in more detail in Section 3.3.2. The dysfunction of mitochondria and the ER stress induced by impaired lipid metabolism contributes to the generation of ROS, thus causing OxS. The antioxidant balance is further compromised by downregulation of the antioxidant response (e.g., the Nrf2 pathway) and by decreased levels of antioxidant enzymes and molecules (e.g., GSH and SOD). 

### 3.1. Lipid Metabolism and Oxidative Stress

In physiological conditions, lipid homeostasis in the liver is maintained by fatty acid uptake (dietary source) and synthesis of triglycerides (TG) versus the catabolic processes involving oxidation and secretion of lipids. Non-esterified fatty acids (NEFAs) can be used as a source of energy, but they can also be esterified into triglycerides either for lipid storage or VLDL synthesis and secretion into the blood. Fatty acid synthesis or de novo lipogenesis occurs in the cytosol, involving several enzymatic pathways. Fatty acid oxidation takes place mainly in mitochondria and to a lesser extent in peroxisomes and in the endoplasmic reticulum (microsomes) [52]. 

Indeed, it has been demonstrated that hepatic oxidative stress and inflammation are associated with an elevated oxidative metabolism of saturated fatty acids (SFAs) in NAFLD [53]. Some saturated long-chain fatty acids are first oxidized in the peroxisomes and then in the mitochondria. However, these long-chain SFAs are often incompletely oxidized in the mitochondria, affecting metabolic activity and leading to ROS overproduction with subsequent mitochondrial dysfunction [2]. 

Fatty acid oxidation depends on internal mitochondrial transport, which is mediated by different mitochondrial membrane proteins (CPTs, CAT, and CACT) and by L-carnitine. Reduced levels of l-carnitine have been reported in NAFLD patients, which may contribute to reduced fatty acid oxidation and subsequent mitochondrial impairment and concomitant ROS production [54]. Supplementation with L-carnitine was shown to be associated with the improvement of liver inflammation and histological parameters in patients with NASH [55]. There is also evidence that L-carnitine supplementation ameliorates steatosis and improves mitochondrial function in the liver by increasing fatty acid oxidation in diabetic mice [56].

OxS can also lead to damage to macromolecules, resulting in the formation of toxic products. For example, lipid oxidation/peroxidation results in the formation of products such as malondialdehyde (MDA), lipid peroxides, 8-isoprostane, and 4-hydroxy-2-nonenal (4-HNE). These molecules are formed by hydroxyl radical attack to fatty acyl chains of phospholipids and triglycerides. Lipid peroxidation may lead to the downstream generation of reactive molecules (aldehydes) and/or the impairment of cellular structures and architecture, e.g., via structural changes in cellular membranes. In summary, OxS plays a pivotal role in NAFLD pathophysiology, which is linked to impaired lipid metabolism. Therefore, OxS and its downstream impairment of lipid metabolism are valid targets for NAFLD therapy. 

### 3.2. Lipotoxicity in Non-Alcoholic Fatty Liver Disease

As already mentioned, OxS is linked to the pathogenesis of NAFLD, and this has been observed in experimental models of NAFLD/NASH [9] as well as in NAFLD patients who have an impaired redox balance, demonstrated by decreased levels of hepatic glutathione and anti-oxidant enzymes such as SOD and catalase [5,57]. The origin of the impaired redox status in NAFLD can be traced back to impaired lipid metabolism in the liver, resulting in an increased FFA pool in the hepatocytes [58]. This is due to an increased FFA uptake from dietary sources, increased de novo lipogenesis [59], and enhanced lipolysis in adipose tissue, which increases FFA delivery to the liver [60,61]. This excess FFA initially increases mitochondrial β-oxidation as an adaptive mechanism [53], which increases ROS production, but extramitochondrial oxidation is also enhanced [62], resulting in ROS overproduction, which then impairs the antioxidant balance and triggers lipotoxicity in the liver. Excess FFAs can also be incorporated as triglycerides in lipid droplets, causing steatosis [52]. Palmitate is the most abundant saturated long-chain fatty acid present in the diet, and its accumulation leads to increased levels of diacylglycerols (DAGs) and ceramides [63], both considered as lipotoxic lipid species, causing mitochondrial dysfunction and ER stress [48]. These toxic lipid species can induce lipotoxic effects via both direct and indirect mechanisms. For example, ceramides cause the impairment of mitochondrial function via inhibition of β-oxidation and increasing ROS production inside the mitochondria. In addition, DAG and ceramides activate several signalling pathways, including the proinflammatory NF-KB pathway and the NLRP3 inflammasome, as well as the JNK cell death signalling pathway [48]. These phenomena promote cellular dysfunction and further promote cell death and inflammation [64], in addition to impairing the antioxidant balance in hepatocytes. Altogether, mitochondria and ER contribute to the majority of cellular ROS production. 

It has been demonstrated that FFA toxicity can be reduced by its incorporation into triglycerides and lipid droplets [65,66]. In fact, there is a growing body of evidence suggesting that hepatic TG accumulation is not itself harmful for hepatocytes, but rather a protective mechanism against lipotoxicity and consequently excessive ROS production [67,68]. Studies in experimental models of NAFLD also demonstrated that liver injury is caused by FFAs rather than TG accumulation. In fact, FFAs and their metabolites are known to induce hepatocyte injury by increasing OxS [69]. On the other hand, it has been demonstrated that not all species of FFAs are toxic to hepatocytes: MUFAs induce lipid droplet accumulation in vitro without affecting cell viability. In contrast, SFAs, such as palmitate, are toxic and induce only minimal changes in lipid droplet accumulation [70]. Therefore, saturated fatty acids are considered harmful to the cells compared to non-saturated fatty acids (e.g., MUFAs). In fact, the toxic effects of SFAs can be partially abolished by MUFAs, most likely through downregulation of proapoptotic pathways and favouring incorporation of SFAs into TG [64,70].

Parenchymal cells, e.g., hepatocytes, are the main cells affected by lipotoxicity-induced OxS in the liver. However, non-parenchymal cells (NPCs), including Kupffer cells, hepatic stellate cells (HSCs), and liver sinusoidal endothelial cells (LSECs), are also targets of OxS but may, compared with hepatocytes, respond in different ways [71]. Specifically, OxS promotes M1 polarization and activates inflammatory pathways in Kupffer cells, leading to an increased release of pro-inflammatory cytokines such as TNF-α. HSCs can be activated by lipid peroxidation and OxS, promoting their fibrogenic phenotype, resulting in increased synthesis of extracellular matrix components such as collagen [72]. Finally, OxS can damage LSECs. Thus, OxS induced by impaired lipid metabolism can directly kill cells (hepatocytes or LSECs), promote inflammation via activation of Kupffer cells, and promote fibrogenesis via activation of HSC. Extracellular vesicles and/or soluble factors from steatotic or injured hepatocytes/LSECs may aggravate the inflammatory response [73]. It is, therefore, important to also consider the role of NPCs in the pathogenesis of NAFLD and in the development of novel therapeutic targets. 

It has been reported that the gut microbiota and the liver—gut axis play an important role in NAFLD [74]. This interaction is bi-directional: obesity and the increased dietary intake of (saturated) fats as well as the resulting changes in lipid metabolism cause profound changes in the composition of the gut microbiota [75]. On the other hand, changes in the microbiota can aggravate metabolic disturbances and NAFLD [76]. Dysbiosis is defined as changes in the microbiota that have detrimental effects. It has been demonstrated that the transfer of microbiota from obese to lean mice induces metabolic alterations in the recipient mice that are similar to those observed in the (obese) donor mice. It has also been demonstrated that mice with transferred gut microbiota from calorie-restricted mice show resistance to obesity and hepatic lipid accumulation when challenged with an HFD [77], which can be explained in part by the role of gut microbiota in the metabolism of PUFAs from dietary sources [78]. Dysbiosis induces changes in the synthesis of short chain fatty acids (scFFAs) by gut bacteria. These scFFAs act as ligands for G-protein coupled receptors (e.g., GPCR41 and GPCR43) that are involved in the pathogenesis of NAFLD. Dysbiosis also leads to decreased synthesis of Fasting-Induced Adipocyte Factor (FIAF). FIAF inhibits lipoprotein lipase, stimulating the release of free fatty acids [79]. The excess FFAs can subsequently disturb mitochondrial metabolism and lead to increased generation of ROS. Finally, dysbiosis increases intestinal permeability, leading to the translocation of bacteria to the liver, resulting in increased generation of ROS and exacerbating the inflammatory response [74]. Bacterial endotoxin or lipopolysaccharide (LPS) cause the activation of Kupffer cells through interaction with Toll-like receptors (e.g., TLR-4), which leads to the release of proinflammatory cytokines (e.g., TNFα) and free radicals [80]. Moreover, it has been demonstrated that palmitate induces inflammation and macrophage infiltration in the liver, and the subsequent palmitate-induced liver injury is exacerbated by a gut-derived endotoxin [81]. Since dysbiosis is strongly involved in the pathogenesis of NAFLD, the microbiota (microbiota transfer) may also be considered as a target in the therapy of NAFLD.

### 3.3. Cellular Dysfunction Triggered by Fatty Acids in the Liver

#### 3.3.1. Mitochondrial Dysfunction 

Mitochondria are important organelles in hepatic lipid metabolism and are involved in different metabolic processes, such as the transport and oxidation of free fatty acids, and they play a critical role in maintaining a balanced lipid metabolism. In NAFLD, especially at the NASH stage, mitochondrial damage can be observed, e.g., as structural changes, characterized by a rounded morphology and the loss of discernible cristae structures [82]. Functionally, an impaired mitochondrial respiratory chain (MRC) activity with reduced ATP production is observed, although the stage at which the mitochondrial oxidation is impaired is not yet known [2,83,84,85]. At the early stage of NAFLD, the increased lipid flux leads to enhanced but also incomplete mitochondrial oxidation, which aggravates ROS production and generates toxic lipid intermediates [58,83]. Mitochondria are a major source of pro-oxidants and generate superoxide anions via complex I, II, and III of the MRC and hydrogen peroxide via the action of superoxide dismutase. The electron leakage from MRC can be partially compensated by the induction of uncoupling proteins (UCPs), which dissipate the proton gradient, thus leading to reduced ROS generation. However, uncoupling also further reduces ATP synthesis [86]. Furthermore, the reduced levels of antioxidants also contribute to the accumulation of ROS in mitochondria. Mitochondrial coenzyme Q10, which functions as an antioxidant in the mitochondrial membrane, is reduced in NASH patients [9]. The consequences of oxidative stress are not limited to only an impaired redox status, but also the impairment of specific signalling pathways, e.g., the deactivation of phosphatases [87].

Increased levels of (toxic) lipid intermediates as well as lipid peroxidation are additional mechanisms of mitochondrial dysfunction in NASH. Several types of lipid metabolites are increased in NAFLD, including glycerophospholipids, sphingolipids, and free cholesterol [88]. These toxic lipids and lipid intermediates that are generated in lipid metabolism hamper mitochondrial respiration and can disrupt signalling pathways. Moreover, cardiolipin, a type of phospholipid located in the mitochondrial inner membrane and unsaturated fatty acids are particularly susceptible to ROS attack, and these oxidized lipids have indeed been demonstrated in NAFLD. The oxidation of these lipids can alter the structure of the mitochondrial bilayer, membrane fluidity, and permeability and potentiate the proton leak and cytochrome c release, further deteriorating mitochondrial function and inducing apoptosis [89,90]. The deteriorated mitochondrial function can also aggravate the production of ROS, thus constituting a vicious cycle that exacerbates mitochondrial dysfunction and oxidative stress.

In addition to overproduction of ROS and toxic lipids, reduced mitochondrial DNA (mtDNA) content and the changes in mitochondrial biogenesis have also been observed in NASH [91]. NAFLD patients, especially at advanced stages (NASH), demonstrate decreased mtDNA content/copy numbers, accompanied by an increased mutation rate and increased degree of heteroplasmy, which causes mutations in mtDNA encoded genes such as the components of the OxPhos chain. Moreover, the leakage of mtDNA from mitochondria can activate several cellular receptors (e.g., Toll-like receptor-9) and initiate their downstream signalling pathways [92]. Additionally, NAFLD patients demonstrate increased mitochondrial biogenesis and mass but defective mitophagy, which correlates with disease severity [82,89]. Peroxisome proliferator-activated receptor coactivator 1 (PGC1α) and nuclear respiratory factor 1 (NRF1) are two critical regulators of mitochondrial biogenesis that regulate the key factors in mitochondrial oxidation and oxidative phosphorylation. In advanced NAFLD stages, e.g., NASH, the increased mitochondrial mass is accompanied by reduced mitochondrial metabolic capacity, which indicates defective mitophagy and implies that impaired mitochondrial biogenesis might be secondary to other causes of mitochondrial dysfunction as described above. 

The balance between oxidants and antioxidants plays a pivotal role in the maintenance of mitochondrial function during the progression of NAFLD (Figure 1). Supplementation of antioxidants (e.g., MitoQ, coenzyme Q10, and chemical ROS scavengers) can efficiently eliminate ROS, thus preserving mitochondrial function [93]. Moreover, targeting antioxidants to mitochondria seems to be a promising therapeutic option in OxS-related diseases [94,95]. In addition, the inhibition of anaplerosis, the metabolic reactions that generate TCA cycle intermediates, by knockdown of phosphoenolpyruvate carboxykinase 1 (PCK1) also showed protection against hepatic oxidative stress, inflammation, and insulin resistance [53]. Moreover, several studies have shown that inhibiting mitochondrial β-oxidation or suppressing mitochondrial respiration can significantly counteract HFD-induced oxidative stress, hepatic steatosis, and insulin resistance. Liver-specific knockout of mitochondrial long-chain fatty acid transport protein (carnitine palmitoyltransferase 2, CPT2) [96], inhibition of the mitochondrial ATP transporter (adenine nucleotide translocase 2, ANT2) [97], and deletion of the mitochondrial flavoprotein apoptosis-inducing factor (AIF) [98] have each been shown to ameliorate NAFLD. All in all, these results suggest that correcting mitochondrial β-oxidation or respiration could be a therapeutic strategy in the treatment of NAFLD. However, one study reported that genetically or experimentally induced deficiency of mitochondrial oxidative enzymes (specifically the absence of mitochondrial trifunctional protein, MTP^+/−^) leads to aggravation of hepatic steatosis [99,100]. Therefore, targeting the right mitochondrial molecule is critical in reducing oxidative stress and protection against NAFLD. 

#### 3.3.2. ER Stress

The ER is an important source of ROS (approximately 25% of total cellular ROS production) via the formation of disulphide bonds during protein folding [101]. ER stress is intimately involved in SFA-induced lipotoxicity in the liver and closely related to OxS. Additionally, ER stress is linked to metabolic stress and chronic inflammation through ROS production [74,102].

Recent evidence suggests a strong connection between NAFLD and ER stress via direct and indirect mechanisms [103]. Moreover, ER stress has been shown to increase ROS production during hepatocellular injury [104]. Three specific mechanisms through ER stress activate cellular stress and cell death, eventually leading to the activation of C/EBPα-homologous protein (CHOP) and c-Jun N-terminal kinase (JNK), increasing ROS production, which directly affects TNF-α and NF-κB activation and the disruption of calcium homeostasis [105]. 

ROS are generated in the ER via different mechanisms. First, during protein folding in the ER, protein disulphide isomerase (PDI) activity is dependent on oxidation-reduction reactions, mediated by endoplasmic reticulum oxidoreductin-1 (ERO1), triggering ROS production and affecting ER redox balance [106]. In addition, the ER-specific isoform NADPH oxidase 4 protein (Nox4) is also related to ROS generation via direct interaction with PDI. Therefore, increased Nox4 levels are also associated with increased ROS generation during ER stress [107]. Finally, the microsomal monooxygenase system contributes importantly to ROS production in the ER, via its NADPH-P450 reductase activity [106]. Increased ROS generation in the ER can also be related to glutathione (GSH) depletion [108].

ER stress activates the “Unfolded Protein Response” (UPR), which is initiated by three ER transmembrane proteins: RNA dependent protein kinase-like ER eukaryotic initiation factor-2α kinase (PERK), activating transcription factor 6 (ATF6), and inositol-requiring ER-to-nucleus signalling protein 1 (IRE1α). Initially, the activation of these branches counteracts ER stress, but sustained activation of these pathways leads to a pro-inflammatory response and increased transcription and protein levels of the pro-apoptotic factor CHOP, which mediates the direct transcription of apoptotic BCL-2 protein family members, leading to hepatocyte cell death [102]. 

Regarding the role of ER in lipid metabolism, de novo lipogenesis takes place in the ER, where excess free saturated fatty acids are incorporated into phospholipids of the ER membrane, leading to Ca^2+^ release, mitochondrial permeability impairment, and activation of pro-inflammatory and cell death pathways [109]. The overload of SFAs causes ER stress, disrupting normal ER function and resulting in the accumulation of unfolded and misfolded proteins, which triggers the UPR [108]. During ER stress conditions, the expression of the chaperone Glucose-Regulated Protein 78 (GRP78) is increased, resulting in the activation of the PERK, ATF6, and IRE1 pathways [110]. Feeding–fasting cycles can also cause ER stress and UPR activation, triggered mainly by dietary SFAs (e.g., palmitic acid) as part of normal ER homeostasis. In this context, ER stress and UPR are part of normal lipid metabolism [111]. On the other hand, chronic ER stress induced by impaired lipid metabolism is a key mechanism in several pathological metabolic alterations, including insulin resistance and NAFLD [109]. In these conditions, downstream effectors of ER stress contribute to excessive ROS production and perpetuation of OxS in the liver [105]. 

There is substantial evidence for a role of UPR signalling in the regulation of lipid homeostasis in the liver, and ER stress has also been shown to activate lipogenesis. For example, it has been demonstrated that ER stress can activate transcription dependent on sterol regulatory element-binding proteins (SREBPs) via different mechanisms and induce lipid synthesis [112,113]. SREBP transcription factors are master regulators of hepatic lipid metabolism [114]. In normal conditions, they are bound to ER membranes as inactive precursors. Upon activation, e.g., by low sterol levels, these precursors are cleaved and a water-soluble fragment then translocates to the nucleus [115,116]. Induction of the PERK-p-eIF2α signalling pathway promotes lipid accumulation via SREBP activation [117,118]. It has also been reported that ATF4 and ATF6 are able to activate SREBPs and play an important role in lipid homeostasis during ER stress, mainly by increasing hepatic lipogenesis [119,120,121]. 

In diet-induced obese mice, the overexpression of GRP78 inhibits activation of sterol regulatory element binding protein-1c (SREBP-1c) and subsequent hepatic lipogenesis [122]. In addition, the transcription factor XBP-1, which is activated by IRE1α as part of the UPR response, regulates hepatic lipogenesis by regulating the expression of enzymes involved in lipid synthesis [123]. Hepatic deficiency of XBP-1 in mice is correlated with strongly decreased serum levels of TGs, cholesterol, and free fatty acids and decreased hepatic lipogenesis [124]. Increased hepatic DNL in high-fructose-fed mice is associated with the activation of the two main components of the UPR: IRE1α (IRE1α /XBP1) and PERK (PERK/eIF2α) [125]. The PERK/eIF2α branch is crucial in the regulation of the transcription factors C/EBPα, C/EBPβ, and PPARγ, which control the expression of several lipogenic genes, as previously demonstrated in HFD-fed mice [123]. It has been also demonstrated that ER stress increases the expression of some lipid transporters [126]. 

The ER is the main cellular compartment involved in lipid synthesis and VLDL assembly in hepatocytes. In particular, the IRE1α–XBP1 pathway positively regulates VLDL formation and secretion, and it has been demonstrated that disruption of this pathway in mice (IRE1α-deletion) impairs VLDL-TG assembly, affecting lipid partitioning in the ER lumen without changing TG synthesis or de novo lipogenesis. However, in this study, it was also reported that TG availability in the smooth ER was significantly decreased in these mice, which directly affects VLDL assembly [115,124].

Several experimental models of NAFLD have demonstrated that ER stress is involved in NAFLD-induced liver injury [108]. Ultimately, sustained activation of the UPR can activate JNK, which promotes apoptosis and cell death in NASH [127,128,129,130]. SFAs cause apoptosis via the induction of ER stress. Specifically, palmitate can induce apoptosis via the IRE1, PERK, and ATF6 pathways, and in vitro models using primary β-cells showed that knockdown of PERK significantly attenuates palmitate-induced cell death. Moreover, palmitate activates JNK and inhibition of JNK reduced palmitate-mediated AP-1 activation. In liver tissue of NAFLD and NASH patients, phosphorylation of eIF2α, an upstream element of the PERK pathway, is increased. In HFD-fed mice, ER stress increases the level of C/EBP-homologous protein (CHOP), an important component of the UPR pathway [131]. 

It has been suggested that, in conditions of metabolic stress, lipotoxicity is related to excessive incorporation of SFAs into the ER membranes, reducing the presence of other types of lipids such as sphingomyelin and cholesterol [102]. In addition, SFA overload in the ER increases the ratio of phosphatidylcholine (PC)/phosphatidylethanolamine (PE), resulting in the inhibition of Sarco/ER Calcium ATPase (SERCA) activity and disrupting ER calcium homeostasis. The ER is characterized by a very high calcium (Ca^2+^) concentration in physiological conditions, which is maintained by the SERCA ATPase. This regulation is crucial for ER function, because many chaperone proteins and enzymes involved in protein folding and maturation processes are dependent on these high Ca^2+^ levels [105]. Furthermore, the phosphorylation of IRE1α and eIF2α as well as the expression of GRP78 and GRP94 are changed, indicating activation of the UPR [132]. 

Based on recent evidence and the critical role of ER stress mechanisms in the pathophysiology of NAFLD, ER stress should be considered as an important target for novel therapeutic approaches.

## 4. Antioxidants as a Therapy in NAFLD 

**Figure 2:**
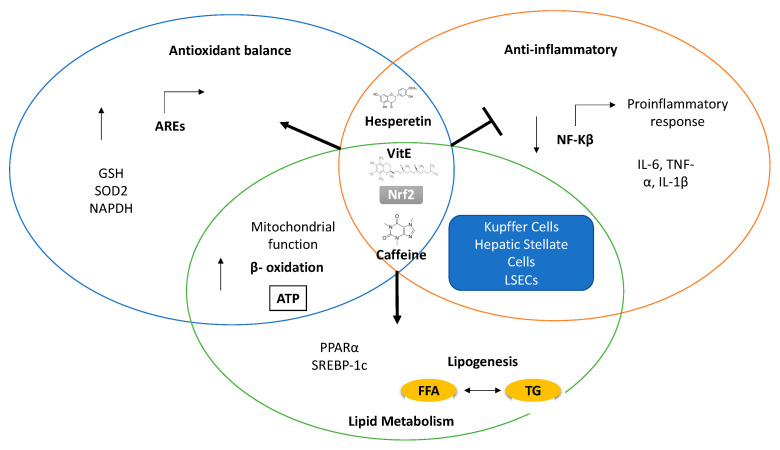
Potential therapeutic strategies for NAFLD based on antioxidant potential. Main mechanisms related to the potential therapeutic effect of antioxidant agents in NAFLD, according to experimental evidence. During NAFLD progression, ROS overproduction leads to an impairment of the antioxidant balance and lipid metabolism and to a proinflammatory response, resulting in liver injury. Therapeutic antioxidant effects are mediated by the activation of Antioxidant Response Elements (AREs) regulated by the Nrf2 and NF-kB pathways. Restoration of mitochondrial function normalizes lipid metabolism by restoring the balance between lipolysis and triglyceride synthesis and improves β-oxidation and ATP generation. Finally, anti-inflammatory effects, mainly via inhibition of NF-κB-dependent transcription, prevent liver injury and non-alcoholic steatohepatitis (NASH) progression.

As discussed above, lipotoxicity plays a central role in the progression of NAFLD. Lipotoxicity is closely related to the redox balance and OxS in the liver, and new therapeutic targets aimed at improving the redox balance are being investigated (Figure 2). 

Clinical evidence demonstrates that dietary supplementation with different types of polyphenols significantly improves NAFLD prognosis in individuals at high cardiometabolic risk [133,134]. These data suggest that the antioxidant effect of polyphenols might be beneficial against hepatic fat accumulation, liver inflammation, and fibrosis. Furthermore, various signalling pathways have been described to explain the therapeutic effects of antioxidants such as polyphenols in NAFLD [135]. Activation of the Nrf2 pathway and attenuation of NF-kB signalling are the most investigated. Moreover, natural dietary antioxidants may also contribute to improve some OxS-related phenomena, such as impaired lipogenesis, mitochondrial dysfunction, insulin resistance, and inflammation [136,137]. 

Nrf2 activators, either natural or synthetic compounds, have been proposed as potential therapy, and some of these (e.g., Oltipraz) have been evaluated to assess their therapeutic effect in NAFLD patients. However, due to their electrophilic nature, some side effects are predicted, and more specific, less electrophilic ones are also in development. Natural compounds such as resveratrol and quercetin have also been reported as Nrf2 modulators and as candidates for treatment of NAFLD [138]. However, Nrf2-independent mechanisms, related to the protection against NAFLD in HFD mice, have also been described for antioxidant molecules, for example, compounds present in green tea [38,139]. Some experimental evidence from in vitro models suggests that antioxidants can prevent lipid accumulation via different molecular mechanisms, such as AMPK phosphorylation [140]. 

Clinical and experimental evidence links OxS to NAFLD, and there is evidence that antioxidants can protect against NAFLD/NASH [141]. It is known that many natural compounds exhibit in vitro and in vivo antioxidant effects. Moreover, some of these compounds have been shown to have a therapeutic effect in different liver diseases, including NAFLD. However, in many of these cases, the mechanism of action remains unclear and is not always related to antioxidant activity [1,142]. In this section, we will discuss some experimental evidence about classical as well as novel antioxidants, including natural compounds and their potential therapeutic effect in NAFLD. Table 1 summarizes the experimental evidence and reported mechanisms for these compounds.

### 4.1. Classical Antioxidants: Vitamin C and Vitamin E 

Vitamin C (VitC) and vitamin E (VitE) are classical antioxidant molecules that protect against oxidative stress by scavenging free radicals. These vitamins have been proposed as a potential therapy in NAFLD, and there is evidence that consumption of VitE/C combinations is beneficial in NAFLD/NASH [141]. Moreover, it has been reported that dietary intake of VitE and VitC is inversely associated with the severity of NAFLD [143]. VitE/C supplementation has been reported to improve the antioxidant status of NAFLD patients under statin treatment, but no significant alterations in serum lipid markers were reported [144]. 

VitE is a lipid-soluble vitamin that is normally stored in the liver and fat tissue. It is synthesized by plants as different isoforms, but α-tocopherol is the most common isoform in human plasma and tissues. Tocopherols are direct scavengers of ROS and RNS; however, they also modulate the antioxidant response, increasing the expression of detoxifying enzymes such as SOD, Glutathione Px, and catalase [145]. Furthermore, anti-inflammatory and anti-apoptotic effects have been reported that might be related to the protection against cellular damage [146,147]. VitE treatment slows down NAFLD progression and decreases NAFLD-associated inflammation in clinical trials [148,149], and experimental models have shown that VitE supplementation can efficiently decrease intrahepatic lipid accumulation [150,151]. In fact, several studies demonstrated that both VitE serum levels and VitE/Cholesterol ratio-corrected levels are inversely correlated with the severity of NAFLD [152,153,154]. 

A negative correlation between VitE and VitE/Cholesterol ratio levels and outcome in NAFLD has only been observed in non-diabetic patients. Diabetic patients showed higher levels of VitE probably due to hyperlipidemia, which may lead to higher levels of lipoprotein carriers [155]. In addition, it has been reported that VitE treatment alone only ameliorates steatosis, but was not able to significantly improve the histological outcome in diabetic NASH patients [156]. The explanation for this observation remains unclear, and for this reason, VitE supplementation to treat NASH diabetic patients is not recommended according to current guidelines [157]. 

Whether the protective effect of VitE in NAFLD is related to its antioxidant potential is still a matter of debate. Recent evidence has demonstrated that VitE reduces hepatic DNL via mechanisms dependent on its antioxidant capacity [158], preventing SREBP-1 maturation and translocation to the nucleus. Moreover, the hydroxyl groups and lipophilic side chain of VitE are necessary to observe the inhibition of DNL in HepG2 cells, which suggest that this mechanism is related to its antioxidant activity. 

The VitE antioxidant effect has been related to Nrf2 modulation in different experimental models [159,160,161], but it has not been fully explored in NAFLD models. For example, it has been reported that VitE protects against oxidative stress-related liver injury in rats treated with cadmium, promoting the expression of genes and proteins of the Nrf2 pathway [162]. Furthermore, recent evidence demonstrates that VitE treatment reduces hepatic steatosis in mice with NAFLD induced by fructose feeding, and this therapeutic effect seems to be dependent on the activation of Nrf2/CES1 signalling, which was also observed in in vitro experiments [163]. Further studies are needed to explore the effect of VitE on Nrf2 activation as well as the therapeutic potential of VitE in NAFLD. 

VitC or ascorbic acid is an essential water-soluble vitamin that must be obtained from the diet and cannot be stored in mammals. The therapeutic effect of VitC is not completely clear. Some studies suggest a moderate association between VitC intake and protection against NAFLD, in particular in men and non-obese patients [158]. Other studies have shown a therapeutic effect of VitC in NAFLD. In an experimental model of NAFLD, VitC significantly reduced histological alterations in the liver, reduced hepatic steatosis, and reduced hepatocellular ballooning. However, the NAFLD model used (choline-deficient diet) is not able to reflect liver inflammation and histological features of NASH. Therefore, the value of VitC supplementation in NASH remains unclear [164]. Guinea pigs, like humans, are unable to synthetize VitC. In guinea pigs, an association between VitC deficiency and NAFLD development has been observed [151]. Ascorbic acid supplementation in an HFD model of NAFLD resulted in reduced visceral obesity, inflammation, and apoptosis most likely via increasing the expression of PPARα and genes encoding β-oxidation enzymes [165]. 

VitE/C efficacy studies in NAFLD have demonstrated a clinical response and improvement of biochemical and histological markers [166]. It remains to be elucidated whether the reported beneficial effects of VitE/C in NAFLD patients are related to their antioxidant mechanisms. 

### 4.2. Coffee Components: Caffeine and Coffee Polyphenols

Coffee is among the most consumed beverages in the world. Coffee contains hundreds if not thousands of different compounds. Many of these compounds have been proposed to have beneficial effects on human health [167]. Recent epidemiological studies suggest that coffee consumption protects against different chronic liver diseases, including NAFLD [168,169]. This beneficial effect is partly explained by the presence of caffeine (1,3,7-trimethylxantine), one of the most abundant components in coffee. There is abundant experimental evidence in support of a therapeutic effect of caffeine in the prevention of liver diseases. However, several reports also demonstrated an inverse association between consumption of decaffeinated coffee and liver diseases, suggesting that the protective effect of coffee is partly due to its non-caffeine components [170,171]. 

The molecular mechanisms of the protective effects of coffee are not fully elucidated yet. Evidence suggests a link with the antioxidant response. It has been demonstrated that consumption of coffee increases Nrf2 gene transcription in humans [172]. Experimental models of chronic liver diseases have shown that coffee extracts enhance the antioxidant response and increase the levels of antioxidant enzymes, most likely via modulation of the Nrf2/ARE pathway [173]. 

Moreover, it has been demonstrated that caffeine can regulate lipid metabolism and lipid accumulation in different experimental models [174,175]. Finally, metabolites of caffeine and paraxanthine (1,7-dimethylxanthine) also have antioxidant effects via modulation of the Nrf2 antioxidant pathway [176,177]. Protective effects of caffeine have been reported in different models, and it has been demonstrated that caffeine modulates lipid metabolism [178]. Recent evidence also showed that caffeine treatment promotes the conversion of SFAs to MUFAs [179,180,181]. 

Caffeine has also been shown to improve mitochondrial function and lipid metabolism, in part due to AMPK activation, preventing hepatic steatosis and increasing fat oxidation [179]. Moreover, it has been suggested that the protective effect of caffeine against liver injury might be related to cyclic-AMP related pathways [181,182]. However, the exact molecular mechanisms of caffeine in relation to NAFLD protection still need to be elucidated as well as the potential link with the antioxidant response. 

Coffee is also an important dietary source of natural antioxidants, in particular polyphenols. Among the polyphenols that are present in coffee is chlorogenic acid (CGA). The antioxidant properties of chlorogenic acid have been widely reported. Nevertheless, experimental evidence that the protective effect of coffee components on NAFLD is related to antioxidant effects is still unclear [180,183]. It has been reported that CGA protects against palmitate-induced ER stress and apoptosis in vitro [178,184]. Moreover, based on experimental models of high-fat-/high-carbohydrate-induced NALFD, it has been reported that CGA exhibits hepatoprotective effects, including improvement of metabolic function, reducing inflammation, and improving liver histology [185]. Interestingly, CGA protected against acute liver injury in animal models via activation of the Nrf2 pathway and inhibition of the NLRP3 inflammasome [186,187].

### 4.3. Metformin

Metformin (*N*,*N*-dimethylbiguanide) belongs to the biguanide class of antidiabetic drugs. Metformin is used in the treatment of type 2 diabetes due to its antihyperglycemic effect. T2D is closely associated with NAFLD. Moreover, several studies have demonstrated that metformin treatment slows down NAFLD progression due to amelioration of some metabolic parameters [188,189]. For instance, bodyweight and total body fat mass are significantly decreased after 24 weeks of monotherapy with metformin in T2D patients with NAFLD [190]. However, metformin is still not included in the clinical treatment guidelines for NAFLD [191]. 

More recently, additional mechanisms for the protective effect of metformin in NAFLD have been described, including modulation of intestinal microbiota composition and decreased levels of bacterial endotoxin [192]. There is also evidence showing that metformin can protect against liver damage by lowering the increased serum cholesterol, LDL-c, HDL-c, glucose, ALT, and AST levels in the livers of HFD-fed mice. Metformin alleviates hepatic steatosis by restoring SIRT1-mediated autophagy via an AMP-activated protein kinase-independent pathway. These observations show that autophagy is induced by metformin via the PRKA-mTOR-ULK1 or SIRT1-FOXO signalling pathways. Ob/ob mice, after 4 weeks treatment with metformin, showed increased autophagy that was related to a decreased expression of the signalling adaptor protein SQSTM1/p62 [193]. This protein can be degraded by selective autophagy, and its protein level is often used as an indicator of autophagic flux [194]. 

Interestingly, it has been demonstrated that metformin protects primary rat hepatocytes against oxidative stress-induced apoptosis via the induction of heme-oxygenase-1 (HO-1) and inhibition of JNK activation [195], which suggests a potential therapeutic option for liver diseases associated with OxS. Additional studies have demonstrated that metformin also protects against palmitate-induced necrosis and apoptosis in primary rat hepatocytes and HepG2 cells, respectively. This protective effect is at least in part due to partial inhibition of mitochondrial complex I, leading to increased SOD2 expression and reduced ROS production [196]. Metformin has a beneficial effect on non-alcoholic fatty liver disease induced by an HFD in rats. This beneficial effect includes improved insulin resistance, reduced secreted phospholipase A2 gene expression, reduced secreted phospholipase A2 and lysophosphatidylcholine serum levels, and reduced inflammation, and may be related to improved mitochondrial function [197]. 

Thus, metformin may be useful in the prevention and/or treatment of NAFLD, and its beneficial effects might in part be related to reduced ROS generation in mitochondria.

### 4.4. Hesperetin 

Hesperetin is a flavonoid and a derivative of hesperidin found in citrus fruits such as oranges and grapefruits. Hesperetin has various biological activities, including in vitro and in vivo antioxidant and anti-inflammatory properties. Hesperetin has been shown to attenuate damage induced by hydrogen peroxide via direct radical scavenging and by enhancing the antioxidant response via ERK/Nrf2-mediated induction of HO-1 [198]. In diabetic rats, antioxidant, anti-inflammatory, and anti-angiogenic properties of hesperetin have been reported [199]. Hesperetin significantly attenuated oxidative stress, lipid peroxidation, and ROS production in mouse brains with Aβ-induced neurodegeneration. Furthermore, markers of apoptosis were significantly attenuated by hesperetin in HT22 cells [200]. 

Our previous work demonstrated that hesperetin has hepatoprotective effects against liver injury both in vitro and in vivo [201], and it has also been reported to attenuate hepatic inflammation and fibrosis in vivo [202,203]. Furthermore, a recent clinical study demonstrated that hesperidin (structurally related to hesperetin) improves hepatic steatosis, inflammation, and fibrosis, most likely via modulation of the NF-kB pathway [204]. 

We have also shown that hesperetin protects primary rat hepatocytes and HepG2 cells against palmitate-induced cell death. High concentrations of hesperetin significantly increased GRP78 expression and the level of p-eIF2α [205], indicating that hesperetin may attenuate ER stress. Hesperetin has also been shown to reduce acetaminophen-induced phosphorylation of the MAP kinases p38 and p65, which led to decreased levels of the inflammatory cytokines TNFα and IL-1β [206]. 

Therefore, hesperetin, by improving the redox balance and alleviating ER stress, is a potential therapy that could be used in the treatment of NAFLD. 

## 5. Conclusions and Future Perspectives

NAFLD development and progression involve several mechanisms leading to the disruption of lipid homeostasis. Oxidative stress plays a central role in the pathophysiology of this disease. Tight regulation of the redox balance is essential to maintain lipid homeostasis, in particular to tip the balance towards TG accumulation and away from saturated fatty acid overload within the liver. The imbalance between TG accumulation and SFA overload ultimately triggers lipotoxicity leading to organelle dysfunction, in particular ER stress and mitochondrial impairment. NAFLD is a complex and multifactorial disease in which OxS and lipid metabolism impairment are closely related in a bidirectional way. Although in this review evidence is presented to propose that OxS is among the pivotal mechanisms in NAFLD pathophysiology (based in part on the results of the use of antioxidants in experimental models), this view is still a matter of discussion (e.g., steatosis may also lead to the inflammation and subsequent release of ROS by inflammatory cells). 

Recent experimental evidence shows a potential therapeutic role of the antioxidant response to treat NAFLD, mainly through the regulation of the Nrf2 antioxidant response pathway. Additional hepatoprotective signalling pathways might be involved, although the experimental evidence is still limited. Natural compounds, such as hesperetin and caffeine, are emerging as interesting treatment options for NAFLD, in part because of their potent antioxidant effects and their safety, since many of them are already part of the normal human diet. Indeed, dietary interventions aimed at restoring a “healthy” lipid profile and/or reinforcing the antioxidant status may be important novel strategies for the treatment of NAFLD. Nevertheless, most of the molecular targets and the exact cellular mechanisms of these compounds remain to be fully elucidated, and additional studies on their therapeutic potential are needed. 

## Figures and Tables

**Table 1:** Potential therapeutic compounds based on antioxidant properties.

Antioxidant Compound	Therapeutic Effect	Model	Mechanism	References
Vitamin E	Lipid metabolism improvement; Decrease HSC activation	NAFLD patients	Stabilizing free radicals and prevention against lipid peroxidation. -Nrf2 pathway	[148,149,150,151,152,153,154,155,156,157,158,159,160,161,162,163]
Vitamin C	Suppress HFD-induced visceral obesity	HFD ^1^ C57BL/6J mice NAFLD patients	Increased expression PPARα-dependent fatty acid β-oxidation genes.	[164,165,166]
Caffeine	Reduces hepatic lipid accumulation	Zebrafish HFD ^1^ model	-Nrf2 pathway-ER stress prevention -AMPK activation -Increasing fatty oxidation	[176,177,178,179,180,181,182]
Coffee polyphenols	Reduce hepatic lipid accumulation, proinflammatory cytokine expression. Attenuated fibrosis	Mice models HFD ^1^, MCD ^2^ and CDAA ^3^	Nrf2-ARE pathway activation -Lipogenesis regulation -NLRP3 inflammasome -ER stress and apoptosis protection	[184,185,186,187]
Metformin	protection against palmitate cell death.	Primary Rat hepatocytes HepG2	-Decrease ROS production -Increase SOD expression -mitochondrial restoration -Autophagy induction through AMPK	[188,189,190,191,192,193,194,195,196,197]
Hesperetin	protection against palmitate cell death	Primary Rat Hepatocytes HepG2	-ERK/Nrf2 Induction-ER stress protection -NF-kB modulation	[198,199,200,201,202,203,204,205,206]

^1^ HFD: high fat diet; ^2^ MCD: methionine- and choline-deficient diet; ^3^ CDAA: choline-deficient diet and L-amino-acid-defined diet.

## Abbreviations

OxS	Oxidative stress
ROS	Reactive oxygen species
NAFLD	Non-alcoholic liver disease
NASH	Non-alcoholic steatohepatitis
HFD	High fat diet
ER	Endoplasmic Reticulum
OxPhos	Oxidative phosphorylation
ARE	Antioxidant Response Element
TG	Triglycerides
FFA	Free fatty acids
SFAs	Saturated fatty acids
DAGs	Diacylglycerols
NEFAs	Non-esterified fatty acids
MUFAs	Monounsaturated fatty acids
NPCs	Non-parenchymal cells
LSECs	Liver sinusoidal endothelial cells
HSCs	Hepatic stellate cells
MRC	Mitochondrial respiratory chain
